# Collagen II enrichment through scAAV6-RNAi-mediated inhibition of matrix-metalloproteinases 3 and 13 in degenerative nucleus-pulposus cells degenerative disc disease and biological treatment strategies

**DOI:** 10.3389/ebm.2024.10048

**Published:** 2024-09-02

**Authors:** Demissew Shenegelegn Mern, Claudius Thomé

**Affiliations:** Department of Neurosurgery, Medical University of Innsbruck, Innsbruck, Austria

**Keywords:** intervertebral disc, collagen II degradation in nucleus pulposus, degenerative disc disease, knockdown of MMP3 and MMP13 using scAAV6-RNAi technology, biological regeneration of intervertebral disc

## Abstract

Intervertebral disc (IVD) degeneration damaging the extracellular matrix (ECM) of IVDs is the main cause of spine-associated disorders. Degenerative disc disease (DDD) is a multifaceted disorder, where environmental factors, inflammatory cytokines and catabolic enzymes act together. DDD starts typically due to imbalance between ECM biosynthesis and degradation within IVDs, especially through unbalanced degradation of aggrecan and collagen II in nucleus pulposus (NP). Current treatment approaches are primarily based on conservative or surgical therapies, which are insufficient for biological regeneration. The disintegrins and metalloproteinases with thrombospondin motifs (ADAMTSs) and matrix metalloproteinases (MMPs) are the key proteolytic enzymes for degradation of aggrecan and collagens. Previously, high expression levels of ADAMTS4, ADAMTS5, MMP3 and MMP13, which are accompanied with low levels of aggrecan and collagen II, were demonstrated in degenerative human NP cells. Moreover, self-complementary adeno-associated virus type 6 (scAAV6) mediated inhibitions of ADAMTS4 and ADAMTS5 by RNA-interference (RNAi) could specifically enhance aggrecan level. Thus, MMPs are apparently the main degrading enzymes of collagen II in NP. Furthermore, scAAV6-mediated inhibitions of MMP3 and MMP13 have not yet been investigated. Therefore, we attempted to enhance the level of collagen II in degenerative NP cells by scAAV6-RNAi-mediated inhibitions of MMP3 and MMP13. MRI was used to determine preoperative grading of IVD degeneration in patients. After isolation and culturing of NP cells, cells were transduced with scAAV6-shRNAs targeting MMP3 or MMP13; and analysed by fluorescence microscopy, FACS, MTT assay, RT-qPCR, ELISA and western blotting. scAAV6-shRNRs have no impact on cell viability and proliferation, despite high transduction efficiencies (98.6%) and transduction units (1383 TU/Cell). Combined knockdown of MMP3 (92.8%) and MMP13 (90.9%) resulted in highest enhancement of collagen II (143.2%), whereby treatment effects were significant over 56 days (*p* < 0.001). Conclusively, scAAV6-RNAi-mediated inhibitions of MMP3 and MMP13 help to progress less immunogenic and enduring biological treatments in DDD.

## Impact statement

Degenerative disc disease (DDD) is a common degenerative disorder that starts primarily within the NP. Collagen II, along with aggrecan, is the main and critical ECM components of NP. So far, there are no effective biological treatment strategies available to target the pathological processes of DDD. Current conservative or surgical treatment options, which merely focus on symptomatic interventions, are insufficient for biological restoration of degenerative ECM. Herein we could significantly enhance the level of collagen II in degenerative human NP cells using AAV6-RNAi-mediated inhibitions of MMP3 and MMP13, which are the key degrading enzymes of collagen II in NP. This anti-catabolic biological treatment approach could have a crucial role in suppression of NP degradation and preservation of the ECM. Hence, AAV6-RNAi-mediated inhibitions of MMP3 and MMP13 along with inhibitions of ADAMTS4 and ADAMTS5 enhancing collagen II and aggrecan, could be promising long-term strategies for less immunogenic biological treatments in DDD.

## Introduction

Intervertebral discs (IVDs) contribute to essential functions in terms of load resistance and spinal flexibility. They provide flexibility to the spinal column and resist spinal compression by facilitating uniform load spreading on the vertebral bodies; and act as shock absorber for the spine. IVDs are located between adjacent vertebrae in the spinal column and are composed of three distinct regions. They include the centrally localised nucleus pulposus (NP), the peripherally localised anulus fibrosus (AF) and the cranially and caudally localised cartilaginous endplates (CEP). The highly hydrated NP region of a normal IVD contains dominantly proteoglycan and collagen II, which are considered critical to NP homeostasis. The most abundant proteoglycan in NP is aggrecan, which is responsible for the high water content within the NP. Collage II is crucial in forming a network that holds aggrecan and water together. In addition to collagen II, NP contains to a lesser extent, among other collagen types, also collagen XI, which is vital for the assembly of collagen II fibrils. The fibrocartilage AF region of the normal IVD contains primarily the radially aligned collagen I fibrils and considerably less proteoglycan and collagen II. AF contains to a minor degree, among others collagen types, also collagen XI, which forms crosslinks between the adjacent collagen I fibrils. Collagen I and II constitute about 80% of all collagen types in normal IVDs, whereas under physiological conditions collagen II contains more water than collagen I. The relative proportions of collagen I and collagen II vary incrementally and inversely within an IVD; with almost exclusively collagen I at the outermost layers of the AF and almost exclusively collagen II at the innermost layers near to the NP. The collagen fibrils have an alternating pattern and the thickness of their collagen lamellae varies from 200 to 400 μm, which ascends from innermost layers to outermost layers of the AF. The alternating pattern of collagen fibrils in the AF is designed to resist loading forces exerted on the IVDs and tensile forces encountered during twisting and bending. The compressive loading and the swelling of the hydrated NP can be resisted radially by the collagen fibrils of the AF and axially by the vertebral endplates [1–7]. Degeneration of IVDs in humans, especially lumbar disc degeneration, starts sooner in lifetime than degeneration of other connective tissues. During the progression of IVD degeneration the structural integrity and the load-bearing ability decreases gradually, while the dynamic load transfer to adjacent vertebral bodies increases permanently. Thereby damages in the endplates and trabecular bones can occur, and formation of osteophytes can emerge. Moreover, persistent and increasing loading exerted on the facet complex may cause accelerated arthrosis and neural impingement [3, 4]. Lack of structural integrity in the lamellae of the AF (degenerative fissures) can lead to herniation of the NP and cause disc associated pain in some patients. Degeneration of IVDs that results in painful arthritis, disc herniation or spinal stenosis is referred to as degenerative disc disease (DDD).

DDD is one of the most common musculoskeletal disorders and a critical contributor to chronic back pain. It substantially affects the quality of life in terms of disability, social isolation and huge medication costs [8–14]. However, current treatment options are based on either conservative therapies or surgical methods, which can remove only the symptomatic tissue and reconstruct the segment through surgical fusion or disc arthroplasty [15–18]. They do not address the essential biological restoration of the disc tissues. Therefore, options of biological therapies that can restore or regenerate the damaged IVDs are crucial.

Accelerated DDD involving loss of structural integrity can be caused by different factors, especially by genetic predisposition and environmental influences that lead to imbalanced anabolism and catabolism within IVDs. These include, such as early cellular senescence, decreased ECM production, increased expression of inflammatory cytokines and enhanced activities of degradative enzymes within the avascular IVDs. Cells within IVDs generally acquire nutrients via diffusion through capillaries of CEPs and survive in the oxygen deprived slightly acidic environment. However, with aging the narrowing of the blood vessels near the disc-bone junction of the vertebral body or calcification of the CEPs affect the diffusion of nutrients, and promote more stressful acidic microenvironment within the IVDs. This nutritional deprivation may turn the balance toward accelerated IVD degeneration through early cellular senescence, attenuated ECM formation (anabolism) and increased ECM degradation (catabolism) [19–30].

NP cells play a key role in IVD homeostasis by orchestrating the expressions and activities of anabolic, catabolic, anti-catabolic and inflammatory factors. The degree of imbalances between ECM formation and ECM degradation is highly correlated with the grade of IVD degeneration. High-grade degeneration leading to diminished level of aggrecan and collagen II crucially deteriorate the proper function of IVDs, particularly within the NP. Deregulations of the normal homeostatic mechanism have been repeatedly shown in degenerative NP cells. These involve increased accumulation of selective inflammatory factors (such as TNF-α and IL-1β) and catabolic factors (such as ADAMTSs and MMPs), along with low levels of their natural inhibitors (such as TIMPs) and anabolic factors (such as BMPs, TGF- β and IGF-1) [31–33]. High expression levels ADAMTS4 and ADAMTS5 together with high expression levels of MMP3 and MMP13, which were allied with low levels of aggrecan and collagen II, have been shown in our previous studies of degenerative NP cells [31, 32]. Furthermore, we recently demonstrated that scAAV6-mediated specific inhibition of ADAMTS4 and ADAMTS5 by RNA-interference (RNAi) could enhance the level of aggrecan, without affecting the level of collagen II [34–36]. These findings suggest that MMPs are evidently the main degrading enzymes of collagen II in NP. Therefore, in this study we investigated scAAV6-mediated specific knockdown of MMP3 and MMP13 in degenerative NP cells. Suppression of MMP3 and MMP13 using RNAi-mechanism leading to enhancement of collage II level, without any impact on the level of aggrecan, would be one of the significant therapeutic approaches for inhibition of DDD. Accordingly, developing combinatorial therapeutic strategies, which are able to suppress the designated ADAMTSs and MMPs in NP cells, are crucial for biological restoration of degenerative IVDs.

## Materials and methods

### Ethical considerations in recruiting samples from patients

**FIGURE 1:**
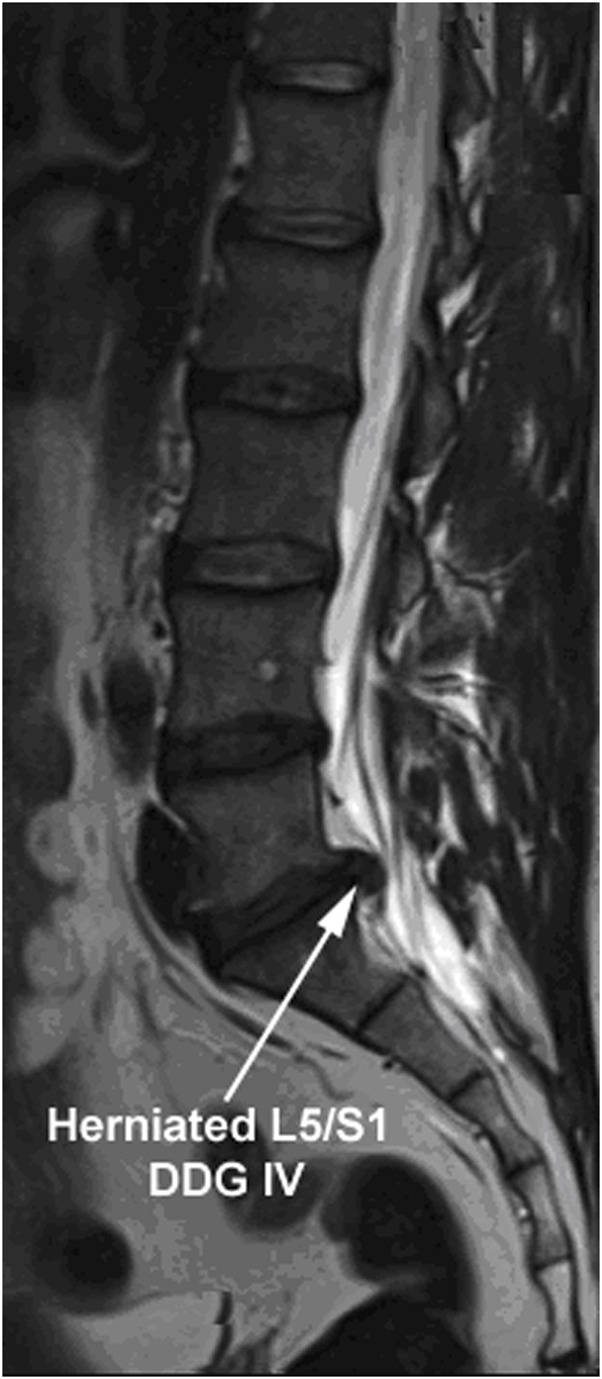
T2-weighted MRI image representing the herniated IVDs of DDG IV. T2-weighted MRI image demonstrating a herniated lumbar IVD (L5/S1) of disc degeneration grade IV (DDG IV) with nerve root compression that remained unresponsive to non-operative treatment for 6 weeks. Sixteen patients harboring a herniated lumbar IVD of DDG IV were involved in this study (age range: 40–69 years, mean age: 55 years). The MRI based interobserver reliability agreement for rating of DDG was ascertained with 100% frequency of agreement (κ = 1.00).

The local research ethics committee authorized this experimental study (Medical University of Innsbruck: project AN2014-0027 333/4.24). IVD tissues were recruited from patients during lumbar disc surgery. Patients provided their written informed consent to participate in this study. The inclusion criteria for surgery were lumbar disc herniation with nerve root compression detected on MRI, which correlated to primary symptoms that remained unresponsive to non-operative treatment for 6 weeks, or demonstrated progressive neurological deterioration in the face of conservative treatment. Samples from sixteen lumbar discs of sixteen patients, which showed Pfirman disc degeneration grade IV (DDG IV) on MRI, were included in this study [37, 38]. The samples recruited from the sixteen patients are presented in Table 1 (age range: 40–69 years, mean age: 55 years). To determine the DDG of patients, preoperative T2-weighted MRI of the spine was applied. An example of the samples showing a herniated lumbar IVD of DDG IV with nerve root compression is illustrated by using T2-weighted MRI in Figure 1. Lumbar IVD tissues were recruited from the NP sector by nucleotomy and swiftly brought to the laboratory in sterile phosphate buffered saline solution (PBS) for immediate cell isolation.

**TABLE 1:** IVD samples recruited from sixteen patients showing lumbar disc levels and lumbar disc degeneration grade (DDG) with age and sex.

Sample	Disc level	DDG	Age/Sex
1	L4/L5	IV	40/F
2	L4/L5	IV	42/F
3	L4/L5	IV	43/M
4	L5/S1	IV	46/M
5	L5/S1	IV	48/M
6	L4/L5	IV	50/F
7	L4/L5	IV	52/F
8	L5/S1	IV	55/M
9	L5/S1	IV	58/F
10	L4/L5	IV	59/M
11	L5/S1	IV	60/M
12	L5/S1	IV	63/F
13	L4/L5	IV	64/M
14	L5/S1	IV	65/F
15	L5/S1	IV	66/F
16	L4/L5	IV	69/M

### NP cell isolation and monolayer expansion

IVD tissues that were recruited from patients were immediately brought to the laboratory in PBS and washed at 1,000×g for 2 min. The NP tissues of the IVDs were separated from the AF residual based on their macroscopic morphology (identification of the innermost lamellar rings of the AF). The isolated NP tissues were finely chopped up into small fragments and digested with pronase (0.02% w/v, 37°C, 5% CO_2_, 1 h) in 20 mL DMEM (Dulbecco’s Modified Eagle’s Medium) culture medium, which contained 1% penicillin/streptomycin, 1% glucose and 10% FBS (fetal bovin serum) (Sigma-Aldrich). The samples were then filtered through sterile 75 gm nylon mesh filters (Sigma-Aldrich). The supernatants were centrifuged at 1,000×g for 2 min. Pellets were suspended in 20 mL DMEM and redigested with collagenase II (0.02% w/v) and hyaluronidase (100U) (Sigma-Aldrich) for 180 min (37°C, 5% CO_2_). The redigested samples were filtered through sterile 75 gm nylon mesh filters and supernatants were centrifuged at 1,000×g for 2 min. The pellets were resuspended in DMEM culture medium of 10 mL and cultured in 25-cm^2^ tissue culture flask (Sigma-Aldrich) for 2 weeks (37°C, 5% CO_2_) by changing the culture medium every 2 days. Proliferated NP cells at the confluent of 100% were split in a ratio of 1:2 using trypsin-EDTA (Sigma-Aldrich) for monolayer cell expansion. Expanded NP cells were cryopreserved at −196°C in DMEM culture medium, which contained 30% FBS and 15% dimethyl sulfoxide (DMSO) (Sigma-Aldrich). Cryopreserved NP cells are made available for three-dimensional (3D) cell culture and subsequent analyses.

### Alginate based 3D-culture of NP cells

The Alginate-3D-Cell-Culture Kit (AMSBIO) was used based on the instruction guide of the manufacturer (AMS Biotechnology). Briefly, calcium chloride solution (5 mL) was dispensed in a 10-mL sterile glass cup that contained a sterile stir bar. NP cell pellets were prepared from 1 × 10^5^ scAAV6-transduced or non-transduced (untreated) cells in 1.5-mL Eppendorf-tube by harvesting the cells using trypsin-EDTA after 3 days of transduction. Sodium alginate solution (0.5 mL) was then dispensed into each 1.5-mL tube containing the pellet and mixed with a pipette to make a homogeneous cell suspension. The homogeneous cell suspension in each tube was aspirated into a 1-mL syringe that was attached with a plastic flexible needle. In order to drop the cell suspension into the glass cup containing the calcium chloride solution, the plastic flexible needle was removed and a 22G hypodermic needle was attached to the syringe. The cell suspension was dropped into the stirring calcium chloride solution (5 mL) at about two drops per second by holding the syringe in an upright position over the glass cup, and by positioning the tip of the needle about 5 cm above the liquid surface of calcium chloride. Subsequently the beads in calcium chloride solution were stirred about for 10 min until the alginate beads have coagulated and appeared completely white. The calcium chloride solution was removed using a manual pipette without aspirating the beads and then saline solution (10 mL) was added to the beads. The saline solution was removed after 15 min using a manual pipette, the beads were mixed with culture medium (10 mL) and incubated at room temperature (RT) for 10 min. Right after removing the medium, 10 alginate beads were scooped using a sterile spatula and placed into a separate 24-well plate containing culture medium (2 mL). Cells were cultured in 24-well plates up to 56 days of post-transduction (37°C, 5% CO_2_) by changing the culture medium every 2 days. Three independent 3D-cultures of NP cells were performed for each sample.

### Recovery of NP cells from alginate beads

After 3D-culture of the NP cells in alginate, the culture medium was removed from each well using a manual pipette. The alginate beads embedding the NP cells were then dissolved by adding sodium citrate solution (1 mL) to each well and mixing for 10 min at RT. The entire mixtures in a 24-well plate were transferred to a 50-mL tube and centrifuged at 1,000×g for 2 min. NP cells were harvested as a precipitated pellet and used for subsequent experimental steps.

### Recombinant scAAV6 vector construction

The shuttle plasmid of this study was assembled based on the shuttle scAAV (self-complementary adeno-associated virus) plasmid described before [39, 40]. It contains the expression cassettes of *emerald green fluorescent protein* (emGFP), the shRNA along with the human cytomegalovirus (CMV) promoter and the U6 promoter (a polymerase III promoter). The BLOCK-iT™ RNAi Designer (ThermoFisher) was used to design the shRNAs targeting the mRNAs of MMP3 or MMP13. The corresponding shRNAs were cloned between *BamHI* and *HindIII* restriction sites of the shuttle plasmid. The recombinant shuttle plasmids encoding the shRNAs are termed as plasmid AAV6-MMP3 and plasmid AAV6-MMP13 respectively. The non-target control shRNA is termed as plasmid AAV6-Ctrl. The AAV6 helper plasmid DP6rs was acquired from PlasmidFactory GmbH & Co. KG (Bielefeld, Germany).

The sense sequences of the shRNAs targeting MMP3, targeting MMP13 and the non-target control shRNA are shown below, respectively.

5′- AAT​GGA​GAT​GCC​CAC​TTT​GAT​TTC​AAG​AGA​ATC​AAA​GTG​GGC​ATC​TCC​ATT​TTT​TT-3′

5′- AAG​ACT​TCC​CAG​GAA​TTG​GTG​TTC​AAG​AGA​CAC​CAA​TTC​CTG​GGA​AGT​CTT​TTT​TT-3′

5′- AAT​CTT​ACC​GAG​CAT​GAC​GTT​TTC​AAG​AGA​AAC​GTC​ATG​CTC​GGT​AAG​ATA​TTT​TT-3‘

### Production and purification of the recombinant scAAV6 vectors

For the production of the recombinant scAAV6 vectors the human embryonic kidney 293 (HEK293) cells were grown in DMEM containing 1% penicillin/streptomycin, 4.5% glucose and 10% FCS. Prior to transfection the cells were passaged two times at confluent of 90%. 5 × 10^6^ HEK293 cells were cultured in 15-cm culture dish containing 20 mL culture medium until they reached 70–80% confluent. Initially, 96 µg of the helper plasmid (DP6rs) was mixed separately with a 30 µg of the individual shuttle plasmid AAV6-MMP3 or AAV6-MMP13 or AAV6-Ctrl to prepare the transduction medium. Then each mixture of the individual shuttle plasmid was added to a 2.5 mL of 300 mM calcium phosphate (Sigma-Aldrich) and gently mixed with an additional 2.5 mL of two-fold concentrated HEPES Buffered Saline (2 × HBS) (Sigma-Aldrich). After removing the culture medium from the culture dish and the transduction medium was directly pipetted to the culture dish. After incubation (6 h, 37°C, 5% CO_2_) the transduction medium was replaced by the culture medium containing 2% FCS. At 72 h of transduction the culture medium was collected, cell pellet was harvested by trypsinization and both together were centrifuged for 5 min at 2000xg. The pellet after centrifugation was suspended in a 2.5 mL serum-free DMEM and the sample was subjected to eight rounds of freeze/thaw cycles by alternating the tube between dry ice-ethanol bath and 37°C water bath. The supernatant containing the recombinant scAAV6 vector was collected by centrifugation at 8000xg (30 min) and stored at −80°C for subsequent purification. The recombinant scAAV6 vectors were then purified as previously described [37]. Shortly, the iodixanol (Sigma-Aldrich) gradient centrifugation was used to purify the recombinant scAAV6 vectors from freeze/thaw-supernatants. Then iodixanol was removed by running the iodixanol fractions through PD10 gel filtration columns (GE Healthcare). Ten fractions of each 1 mL eluate were collected and fractions 4 to 6 were pooled for quantification. The quantitative PCR (qPCR) was used to quantify the purified recombinant scAAV6 vectors: AAV6-MMP3, AAV6-MMP13 and AAV6-Ctrl.

### Viral particle quantification of the recombinant scAAV6 vectors

The qPCR was used to quantify the purified recombinant scAAV6 vector particles by using the TaqMan Gene Expression Master Mix (ThermoFisher) and LightCycler 480 (Roche Applied Science). The master mix (1x) supplemented with 200 nM sense and 200 nM antisense primers of 5´-ITR as well as 250 nM ITR-probe and 2 μL of the template DNA was used in the final volume of 20 μL. Hereunder the sequences of the ITR primers and ITR-probe are specified.

5´-ITR-sense: GGA​ACC​CCT​AGT​GAT​GGA​GTT.

5´-ITR-antisense: CGGCCTCAGTGAGCGAG.

*ITR-probe: 6FAM-CACTCCCTCTCTGCGCGCTCG-BHQ1*.

As described before [38] the genomic DNA of the shuttle plasmid was used as a standard and three replicates of the standard, negative control and samples were run in 96 well plate. The applied qPCR program was consisted of an initial denaturation step at 95°C for 10 min followed by 40 cycles with denaturation at 95°C for 15 s and an extension at 60°C for 1 min that included a melt curve stage 65°C–95°C (increment 0.5°C). For the qPCR data analysis, the Applied Biosystems StepOne software v2.1 (Life Technologies) was used and three independent qPCRs were performed for each sample.

### Transduction efficiency evaluation of the recombinant scAAV6 vectors

In 24-well plate, 1 × 10^5^ NP cells per well (about 50% confluent) were seeded and cultured for 24 h in 500 µL DMEM containing 1% FCS (37°C, 5% CO_2_). The recombinant vectors AAV6-MMP3 or AAV6-MMP13 or AAV6-Ctrl at a dose of 5,000 vector genome copy per seeded cell (5,000 vg/c) were used to transduce the cells. Furthermore, NP cells, which were first transduced with AAV6-MMP3 (5,000 vg/c), were additionally transduced with AAV6-MMP13 (5,000 vg/c) after a day of the first transduction, as a combinatorial transduction (AAV6-MMP3 plus AAV6-MMP13). Using fluorescence microscopy (AxioVert.A, Carl Zeiss) the transduction efficiencies were evaluated every 2 days for the first 16 days and weekly up to 56 days. Flow cytometry-assisted cell sorting (FACS) was also used to evaluate the transduction efficiencies in 3D-cultured cells on day 8, 16, 24, 32, 48, 56 after transduction. MoFlo Cell Sorter (Beckman Coulter) was used for FACS analyses in order to count 1 × 10^5^ cells per sample. The number of GFP-positive cells was quantified according to the manufacturer’s protocol. Shortly, the MoFlo Cell Sorter was used with a 100-mm flow cell tip and a flow rate of 12,000 events per second, along with an extension wavelength of 488 nm and a laser power of 110 W. Three independent FACS evaluations with duplicate were performed for each sample.

### Transduction units quantification of the recombinant scAAV6 vectors

The number of recombinant scAAV6 vectors internalized into NP cells (transduction units per cell: TU/Cell) was quantified using the qPCR with the 5′-ITR primers and ITR-probe as described above. NP cells were seeded, transduced, 3D cultured and harvested on day 2, 4, 8, 16, 24, 32, 48 and 56 as defined already. Cell pellets were washed three times with PBS to remove the vectors that remained attached to the surface of NP cells. The washed pellets were resuspended in a final volume of 100 μL and subjected to eight rounds of freeze/thaw cycles by alternating the tube between the dry ice-ethanol bath and the 37°C water bath. Following centrifugation at 17,000×g for 5 min, the supernatants were used for titration of the transduction units per cell (TU/Cell) using qPCR. Untreated NP cells and AAV6-Ctrl transduced cells were used as controls, and the shuttle plasmid was used as standard in 10-fold dilutions from 10^6^–10^3^ copies/μL. Three independent quantifications of TU/Cell were performed for each sample.

### Evaluation of NP cell viability and proliferation rate after transduction

The MTT Assay Kit [3-(4, 5-dimethylthiazolyl-2)-2,5-diphenyltetrazolium bromide assay, ThermoFisher] was used to examine the impact of the recombinant scAAV6 vectors on NP cell viability and growth rate. Before transduction as well as on day 2, 4, 8, 16, 24, 32, 48 and 56 after transduction the viability and proliferation rate of transduced and 3D cultured NP cells were quantified. Cells were seeded, transduced, cultured and harvested as described above. The corresponding untreated NP cells and AAV6-Ctrl treated cells were used as controls. After washing the pellets of NP cells two times with PBS, pellets were resuspended in 250 µL culture medium and duplicates of 100 µL were plated into flat-bottomed 96-well plate. Control wells (only with medium alone) were additionally used to provide the blanks for absorbance readings. 10 μL MTT reagent was added to each well after 24 h incubation for recovering (37°C, 5% CO_2_), and further incubated for 3 h. After adding 100 µL of the SDS-HCl solution to each well, the samples were further incubated for 4 h. To measure the absorbance of the samples at 570 nm, a microtiter plate reader Infinite 200 (TECAN) was used. The average value of the blank duplicate readings was subtracted from the average values of the sample duplicate readings. A standard curve was used to calculate the number of viable cells. Three independent MTT assays with duplicate were performed for each sample.

### Quantification of MMP3 and MMP13 mRNA levels using real-time qRT-PCR

To analyse the impacts of the recombinant scAAV6 vectors (AAV6-MMP3 or AAV6-MMP13 or a combination thereof (AAV6-MMP3 plus pAAV6-MMP13) on the mRNA levels of MMP3 and MMP13, the real-time quantitative reverse transcription PCR (Real-time qRT-PCR) was used. Before transduction as well as on day 2, 4, 8, 16, 24, 32, 48 and 56 after transduction the mRNA levels in transduced and 3D cultured NP cells were quantified. NP cells were seeded, transduced, cultured and harvested as described above. The corresponding untreated NP cells and AAV6-Ctrl treated cells were used as controls. The RNeasy Plus Mini Kit (Qiagen) with DNase I (Sigma-Aldrich) was used to isolate the total RNA without DNA contamination. The amount of total RNA was quantified at 260 nm using a Biospectrometer (Eppendorf). Equal amounts of total RNA were used to perform reverse transcription (RT), and the TaqMan Reverse Transcription Reagents (ThermoFisher) were used to synthesize the cDNAs. The mRNA levels of MMP3 and MMP13 were quantified by real-time qRT-PCRs using the TaqMan gene expression assays and LightCycler 480 as described above, and β-actin was used as internal standard. The data of relative mRNA levels were numerically presented using the comparative 2^−ΔΔCT^ method. Three independent real-time qRT-PCRs with triplicate were performed for each sample. The sense sequences of primers and probes used for the real-time qRT-PCRs are shown below:

MMP3 sense: CTC​ACT​CAC​AGA​CCT​GAC​TC

MMP3 antisense: CTC​AGA​GTG​CTG​ACA​GCA​TC

MMP3 probe: 6FAB-GGCATTCAGTCCCTCTATGG-BHQ1

MMP13 sense: GAA​TTA​AGG​AGC​ATG​GCG​AC

MMP13 antisense: GTC​AAG​ACC​TAA​GGA​GTG​GC

MMP13 probe: 6FAB-GGACAAGTAGTTCCAAAGGC-BHQ1

Beta-actin sense: CAG​AAG​GAC​AGC​TAC​GTG​GG

Beta-actin antisense: CAT​GTC​GTC​CCA​GTT​GGT​CA

Beta-actin probe: 6FAB-GACCCTGAAGTACCCCATCG-BHQ1

### Western blot analysis

To investigate the impacts of knockdowns mediated by the recombinant vectors AAV6-MMP3 or AAV6-MMP13 or the combination of both AAV6-MMP3 plus pAAV6-MMP13, the protein expression levels of MMP3, MMP13 and collagen II were evaluated by western blotting. NP cells were seeded, transduced, cultured and harvested on day 8 and 56 as described above. Untreated and AAV6-Ctrl treated NP cells were used as controls. RIPA buffer (radio-immunoprecipitation assay buffer, Sigma-Aldrich, ice-cold) supplemented with protease and phosphatase inhibitors (Sigma-Aldrich) was used for 20 min (4°C) to lyse the harvested NP cells. Lysed cells were centrifuged for 10 min at 14,000×g (4°C) and supernatants containing the total protein were used for western blotting. The concentration of the total protein was determined using BCA Protein Assay Kit (ThermoFisher). Isolated total proteins were separated based on their molecular weight using SDS-PAGE (sodium dodecyl sulfate polyacrylamide gel electrophoresis) and the separated proteins were transferred to polyvinylidene fluoride (PVDF) membrane (ThermoFisher). Anti-MMP3 antibody (ab53015, Abcam), anti-MMP13 antibody (ab39012, Abcam), anti-collagen II antibody (abx013043, Abbexa) and anti-beta-actin antibody (ab8227, Abcam) produced in rabbits were used as primary antibodies. Goat anti-rabbit IgG H&L horseradish peroxidase-conjugated (HRP) secondary antibody (ab205718, Abcam) and Pierce ECL Plus Western Blotting Substrate (32132X3, ThermoFisher) were used to detect interactions between antigens and primary antibodies on the membrane. The detected protein bands were quantitatively compared using ImageJ. Three independent western blot analyses were performed for each sample.

### Enzyme-linked immunosorbent assays

To investigate the specificities of the MMP3 and MMP13 knockdowns as well as the enhancement of collagen II that are mediated by AAV6-MMP3 or AAV6MMP-13 or a combination of both AAV6-MMP3 plus AAV6-MMP13, enzyme-linked immunosorbent assay (ELISA) was applied. NP cells were seeded, transduced, cultured and harvested on day 8 and 56 after transduction, and total proteins were isolated and quantified as described above. ELISA was performed for each sample with 100 μg of total protein based on the instruction manuals of the kits (R&D Systems, Uscn Life Science Inc.). Untreated and AAV6-Ctrl treated NP cells were used as controls. Beside the protein levels of MMP3, MMP13 and collagen II, the levels of other vital proteins in NP, such as interleukin-1β (IL-1β) and tumour necrosis factor alpha (TNF-α), ADAMTS4, ADAMTS5 and aggrecan were quantified. To measure the absorbance of the samples, a microplate reader Infinite 200 (TECAN) was used at 450 nm with wavelength correction set to 540 nm. The average values of the blank duplicate readings were subtracted from the average values of the sample duplicate readings. Standard curves were used to calculate the protein concentrations in the samples. Three independent ELISA assays with duplicate were performed for each sample.

### Comparative analysis of data

Landis and Koch based interpretations of κ statistics and agreement percentage among two observers were applied to determine the reliability on MRI evaluations of disc degeneration grade [37, 38]. Moreover, the software IBM SPSS Statistics 22 (Armonk, New York, United States) was used for analyses of statistical data. Data of treated and untreated samples were compared by 1-way ANOVA and pairwise comparison. Significance was set at *p* < 0.01. For the relative quantification analyses of the western blot bands the software Image J was used.

## Results

### MRI evaluation of lumbar disc degeneration grade

The rating of lumbar disc degeneration grade (DDG) based on the T2-weighted MRI, which was carried out by Landis and Koch based interpretations of κ statistics and agreement percentage between two observers, resulted in κ = 1.00 (frequency of agreement = 100%).

### qPCR based quantification of recombinant scAAV6 vector genome copy

The recombinant scAAV6 vectors, containing the shRNAs, were produced, purified and quantified as described in the material and method section. 3.5 × 10^7^ HEK293 cells were used for each recombinant vector and using qPCR high final titers (6.4 × 10^10^–2.9 × 10^11^) of vector genome copies were quantified.

### Transduction efficiencies of the recombinant scAAV6 vectors

**FIGURE 2:**
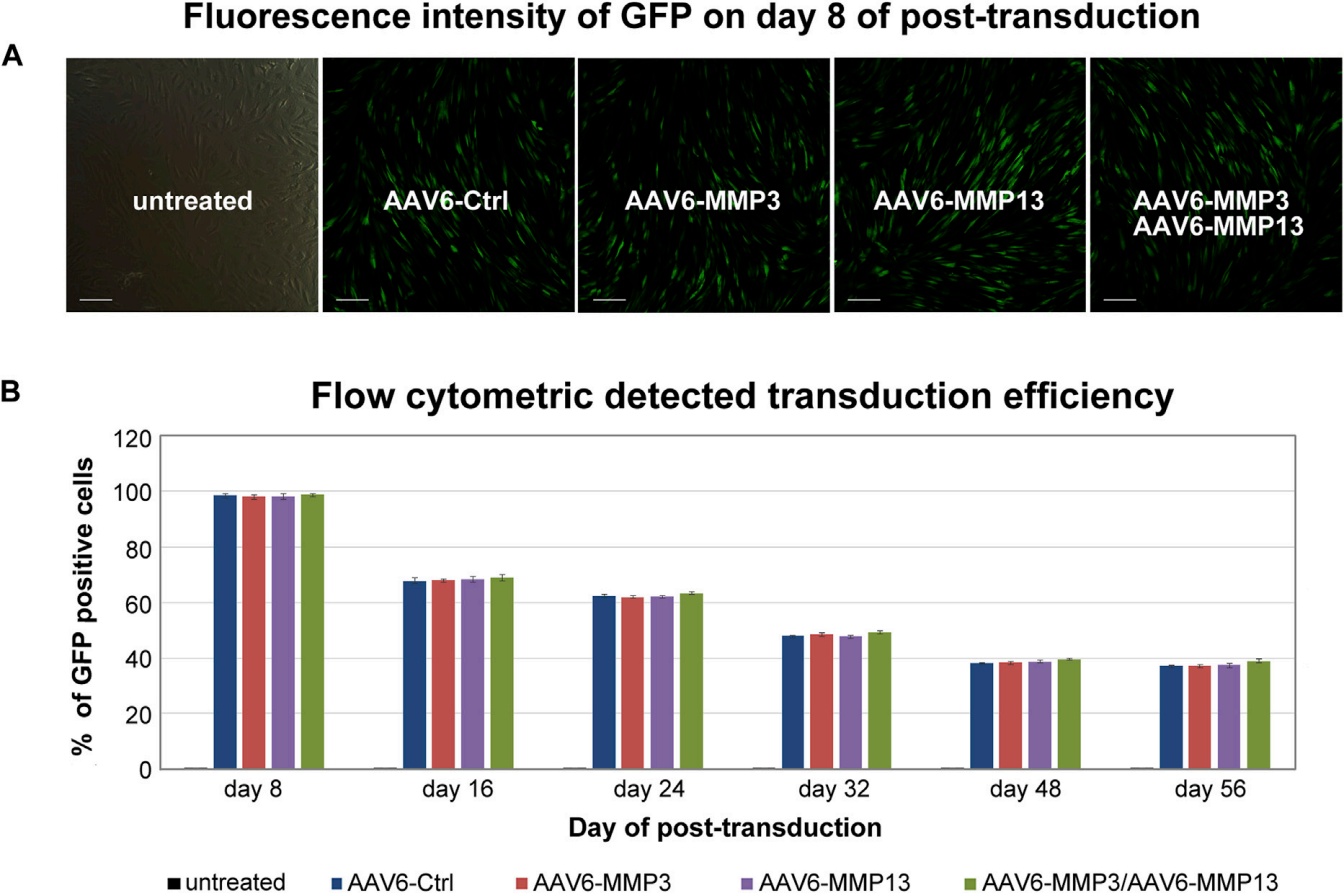
Transduction efficiencies determined by fluorescence microscopy and FACS. After culturing of 1 × 10^5^ cells with DMEM (500 µL containing 1% FBS) in 24-well plate, 5,000 vg/c of each GFP packing recombinant scAAV6 vector were used for transduction. The transductions include 5,000 vg/c AAV6-Ctrl (encoding non-targeting control shRNA), 5,000 vg/c AAV6-MMP3 (encoding shRNA targeting MMP3), 5,000 vg/c AAV6-MMP13 (encoding shRNA targeting MMP13) or 5,000 vg/c AAV6-MMP3 plus 5,000 vg/c AAV6-MMP13. Measurement of transduction efficiencies using fluorescence microscopy and FACS showed similar transduction efficiencies for all applied GFP packing vectors (*p* ≥ 0.315) **(A)**. Measurement of transduction efficiencies using fluorescence microscopy was performed every 2 days for the first 2 weeks and once a week until 56 days. The highest transduction efficiency was detected on day 8 after transduction **(A)**. For precise measurement of the transduction efficiencies, GFP positive cells were counted using FACS on day 8, 16, 24, 32, 48 and 56 by applying 1 × 10^5^ transduced NP cells per sample to the FACS **(B)**. The data represent mean values with standard deviation of three independent experiments.

All recombinant scAAV6 vectors expressing GFP consistently showed similar transduction efficiencies in fluorescence microscopy and in FACS analyses (Figure 2) (*p* ≥ 0.315). The highest transduction efficiencies were verified on day 8 after transduction. The highest transduction efficiencies on day 8, as shown by FACS analyses, amounted to the mean values of 98.35% (98,351 ± 837), 97.91% (97,913 ± 790), 98.14% (98,143 ± 994) and 98.61 (98,611 ± 459) for cells transduced with AAV6-Ctrl or AAV6-MMP3 or AAV6-MMP13 or AAV6-MMP3 plus AAV6-MMP13 respectively. Nevertheless, after day 8 of transduction declining numbers of GFP positive cells were persistently detected during the course of 56 days (Figure 2B). At the end of the FACS analyses on day 56, the transduction efficiencies amounted to the mean values of 37.07% (37,072 ± 432), 37.26% (37,261 ± 542), 37.30% (37,301 ± 710) and 38.09% (38,098 ± 732), respectively. For each sample 1 × 10^5^ NP cells (set as 100%) were used for FACS analyses. Age and gender showed no effect.

### Evaluation of scAAV6 transduction units and cell proliferation rate

**FIGURE 3:**
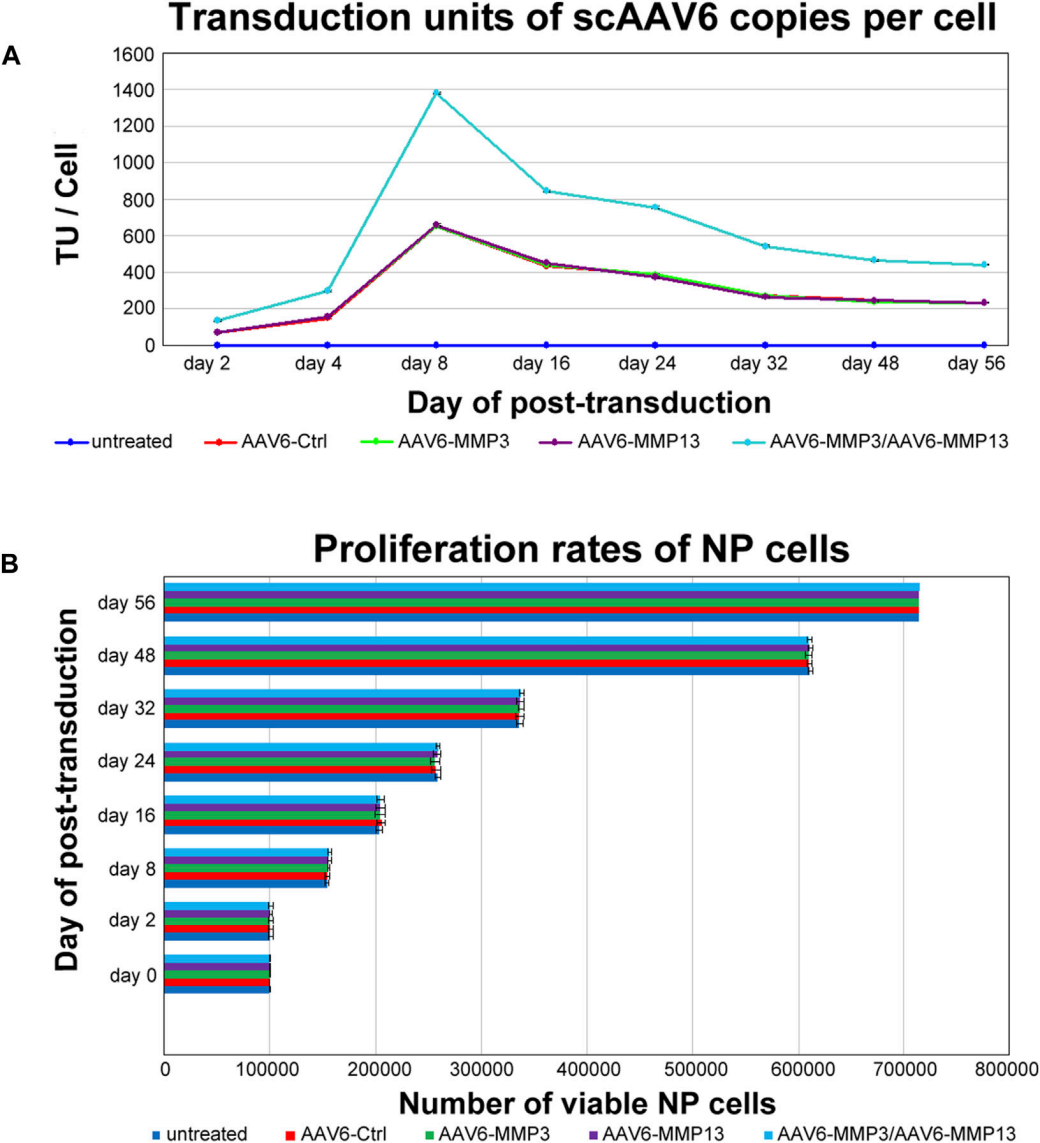
Evaluating transduction units and NP cell proliferation rate. After culturing of 1 × 10^5^ NP cells in 24-well plate, cells were transduced either with 5,000 vg/c AAV6-Ctrl (encoding non-targeting control shRNA), 5,000 vg/c AAV6-MMP3 (encoding shRNA targeting MMP3), 5,000 vg/c AAV6-MMP13 (encoding shRNA targeting MMP13) or 5,000 vg/c AAV6-MMP3 plus 5,000 vg/c AAV6-MMP13. qPCR and MTT assay were used to determine the transduction units per cell (TU/Cell) and cell proliferation rates on day 2, 8, 16, 24, 32, 48 and 56. The qPCR analyses showed similar TU/Cell for each single transduction (*p* ≥ 0.221), but significantly increased TU/Cell for the successive transduction with AAV6-MMP3 plus AAV6-MMP13 (*p* < 0.001) **(A)**. The MTT assays exhibited similar proliferations rates for all untreated and transduced cells (*p* ≥ 0.372) **(B)**. The data represent mean values with standard deviation of three independent experiments.

Determining the number of internalized viral copies per cell, also known as transduction units per cell (TU/Cell), is essential to realize reproducible and practical biological effects, because only a portion of recombinant scAAV6 viral copies, which are attached to the cell surface, are able to get inside the cells. The qPCR was applied to determine the TU/Cell for each recombinant viral vector. The qPCR displayed that transduction with each individual vector constantly reached similar TU/cell (*p* ≥ 0.221), and transduction with combined vectors persistently reached augmented TU/Cell (*p* < 0.001). The maximum TU/Cell was detected within 8 days after transduction. On day 8 the mean transduction units of 652 ± 1.70 TU/Cell, 650 ± 5.47 TU/Cell, 656 ± 5.77 TU/Cell, 1,383 ± 2.80 TU/Cell were determined in AAV6-Ctrl, AAV6-MMP3, AAV6-MMP13 or AAV6-MMP3 plus AAV6-MMP13 transduced cells correspondingly. Nevertheless, the TU/Cell was gradually decreasing after day 8 throughout the 56 days of cell culture. On day 56 the TU/Cell was fallen to the mean values of 231 ± 2.98 TU/Cell, 229 ± 4.50 TU/Cell, 233 ± 3.59 TU/Cell and 439 ± 3.30 TU/Cell correspondingly (Figure 3A). Furthermore, the effects of internalized viral copies on the viability and proliferation rate of NP cells were examined using MTT assays. After seeding of 1 × 10^5^ NP cells in 24-well plate, MTT assays were performed on day 2, 8, 16, 24, 32, 48 and 56 after transduction. Equivalent viabilities and proliferation rates were determined for all transduced and non-transduced cells (*p* ≥ 0.372). Moreover, no effect was observed on the morphology of NP cells. These indicate that the internalized viral copies had no any effect on the viability and proliferation rate of NP cells. After 56 days of cell culture, the mean number of viable cells amounted to 715,269 ± 2,552, 714,684 ± 3,303, 715,164 ± 2,762, 714,298 ± 2,664 and 715,592 ± 2,479 for untreated cells and for AAV6-Ctrl, AAV6-MMP3, AAV6-MMP13 or AAV6-MMP3 plus AAV6-MMP13 transduced cells respectively (Figure 3B).

### MMP3 and MMP13 knockdown and enhancement of collagen II

**FIGURE 4:**
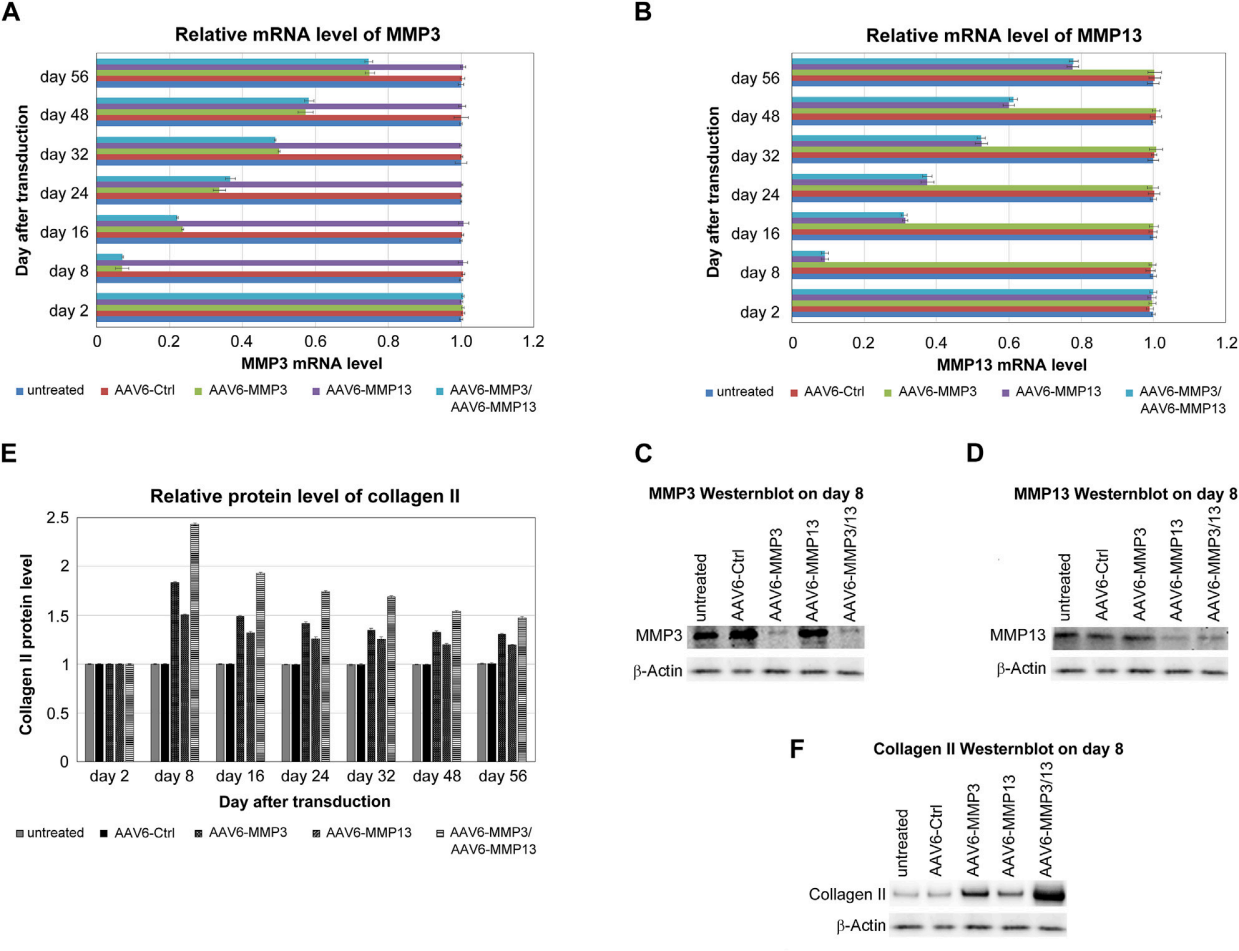
Knockdowns of MMP3 and MMP13 associated with collagen II enhancement. For the transduction of 1 × 10^5^ cells either 5,000 vg/c AAV6-Ctrl (encoding non-targeting control shRNA) or 5,000 vg/c AAV6-MMP3 (encoding shRNA targeting MMP3) or 5,000 vg/c AAV6-MMP13 (encoding shRNA targeting MMP13) or a combination thereof (5,000 vg/c AAV6-MMP3 plus 5,000 vg/c AAV6-MMP13) were used. Real-time qRT-PCR and ELISA were performed after harvesting of transduced cells on day 2, 8, 16, 24, 32, 48 and 56, in order to determine the knockdown impacts on MMP3 and MMP13 in related to collagen II enhancement at mRNA and protein level respectively. The relative mRNA levels of MMP3 **(A)** and MMP13 **(B)** as well as the relative protein levels of collagen II **(E)** are depicted. Western blotting was done to show the maximum knockdowns of MMP3 **(C)** and MMP13 **(D)** as well as the maximum enhancement of collagen II **(F)** on day 8 after transduction. The levels in untreated NP cells, which remained unaffected up to 56 days after transduction, represent the arbitrary unit 1.0. The transductions with AAV6-MMP3 or AAV6-MMP13 or a combination thereof depicted efficient and sustainable knockdowns of MMP3 **(A, C)** and MMP13 **(B, D)** (*p* < 0.001), which are associated with the enhancement collagen II level **(E, F)** (*p* < 0.001). The simultaneous knockdown of MMP3 and MMP13 exhibited additional enhancement of collagen II level. Image J analyses of the western blot bands (ratio: Net Protein X/Net Load Ctrl.) showed knockdown effects of 0.11 for MMP3 and 0.13 for MMP13 as well as enhanced expression effects of 1.92, 1,61 and 2.52 for collagen II in AAV6-MMP3 or AAV6-MMP13 or AAV6-MMP3 plus AAV6-MMP13 treated cells respectively (*p* < 0.001). Untreated and AAV6-Ctrl treated NP cells showed unaffected levels of MMP3, MMP13 (*p* ≥ 0.241) and collagen II (*p* ≥ 0.254). The data represent mean values with standard deviation of three independent experiments.

The recombinant scAAV6 vectors encoding the shRNAs against MMP3 or MMP13 were functionally tested by using qRT-PCR, western blotting and ELISA. The recombinant scAAV6 plasmids encoding shRNAs against MMP3 (AAV6-MMP3) or against MMP13 (AAV6-MMP13) were separately examined. Moreover, both AAV6-MMP3 and AAV6-MMP13 were successively used for simultaneous knockdown of MMP3 and MMP13. The mRNA expression levels of MMP3 and MMP13 were analysed by qRT-PCR up to 56 days after transduction. The highest knockdown impacts on MMP3 and MMP13 were affirmed on day 8 after transduction. On day 8 the average knockdown impacts of 92.9% for MMP3 and 90.6% for MMP13 were confirmed in AAV6-MMP3 or AAV6-MMP13 treated cells respectively. More importantly, similar average knockdown impacts of MMP3 (92.8%) and MMP13 (90.9%) were confirmed after successive treatment of the cells with both AAV6-MMP3 and AAV6-MMP13. Nevertheless, from day 8 until the end of the examination (day 56) the mRNA knockdown efficiencies were steadily declining. Between day 16 and day 56 the knockdown efficiencies were declined to 76.5% and 25% in AAV6-MMP3 transduced cells as well as to 68.6% and 22.3% in AAV6-MMP13 transduced cells respectively (*p* < 0.001). Furthermore, the mRNA knockdown efficiencies in cells transduced with both AAV6-MMP3 and AAV6-MMP13 were similarly declined to 77.8% and 25.4% for MMP3 as well as to 69.9% and 22% for MMP13 respectively (Figures 4A, B) (*p* < 0.001). Additionally, using western blotting the highest knockdown impacts on MMP3 and MMP13 are shown at protein levels (Figures 4C, D). The relative quantification analyses the western blot bands using the software ImageJ showed repression effects of 0.11 for MMP3 and 0.13 for MMP13 as a ratio of the protein expression level in treated cells relative to the expression level of the same protein in untreated cells (ratio: Net Protein X/Net Load Ctrl.) (*p* < 0.001). Untreated and AAV6-Ctrl treated NP cells showed unaffected levels of MMP3 and MMP13 (*p* ≥ 0.241). Furthermore, the impacts of MMP3 and MMP13 knockdown on the enhancement of collagen II were examined using ELISA and western blotting. The results of ELISA also showed that the transduction of NP cells with AAV6-MMP3 or AAV6-MMP13 could enhance the protein levels of collagen II. On day 8 the collagen II levels were enhanced by mean values of 83.4% and 50.2% respectively. Moreover, the successive transduction of NP cells with both AAV6-MMP3 and AAV6-MMP13 showed additive enhancement of collagen II level with the mean value of 143.2% (*p* < 0.001). Nevertheless, the steadily falling knockdown efficiencies of the recombinant vectors on MMP3 and MMP13 during the 56 days of cell culture could also weaken the enhancement of collagen II in a related manner. On day 16 the collagen II levels were enhanced by mean values of just 48.5% in AAV6-MMP3 transduced cells and 32% in AAV6-MMP13 transduced cells, but then by mean value of 92.9% in cells successively transduced with both vectors (*p* < 0.001). On day 56 the collagen II levels were enhanced by mean values of 30.4%, 19.1% and 47.3% respectively (Figure 4E) (*p* < 0.001). Untreated and AAV6-Ctrl treated cells showed unaffected levels of collagen II (*p* ≥ 0.254). The maximum enhancement of collagen II on day 8 after transduction is also shown using western blotting (Figure 4F). The ImageJ ratio analyses (Net Protein X/Net Load Ctrl.) of the western blot bands of collagen II on day 8 showed enhanced expression effects with ratios of 1.92, 1,61 and 2.52 in AAV6-MMP3 or AAV6-MMP13 or AAV6-MMP3 plus AAV6-MMP13 treated cells, respectively (*p* < 0.001). Age and gender showed no effect.

### MMP3/13 knockdown specificity and correlation with collagen II enhancement

**FIGURE 5:**
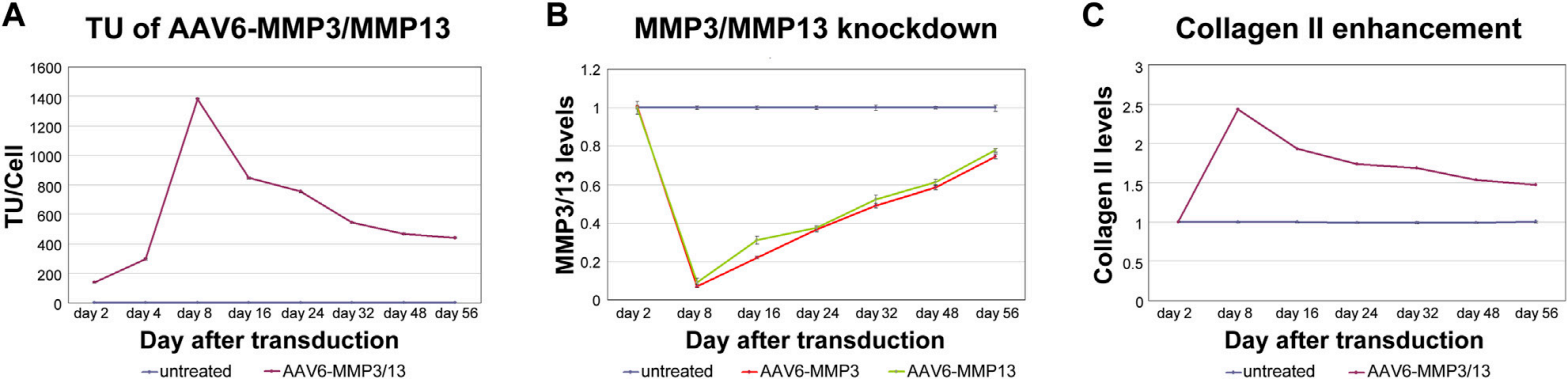
Linking between MMP3/MMP13 knockdowns and collagen II enhancement. 5,000 vg/c AAV6-MMP3 and 5,000 vg/c AAV6-MMP13 that encode the shRNAs targeting MMP3 or MMP13 were used to transduce 1 × 10^5^ NP cells. After transduction, the cells were harvested on day 2, 8, 16, 24, 32, 48 and 56. Then the transduction units per cell (TU/Cell), the mRNA levels of MMP3 and MMP13 as well as the protein level of collage II were determined using qPCR, RT-qPCR and ELISA respectively **(A–C)**. A maximum direct correlation between TU/Cell, knockdown impacts on mRNA and enhancement collagen II protein were depicted in successively with AAV6-MMP3/MMP13 transduced cells. There was no any effect in untreated cells and AAV6-Ctrl treated cells regarding knockdown of MMP3 and MMP13 (*p* ≥ 0.241) or enhancement of collagen II (*p* ≥ 0.254). The levels in untreated cells that remained unaffected up to 56 days represent the arbitrary unit 1.0. The data represent mean values with standard deviation of three independent experiments.

To exhibit the direct correlations between the numbers of internalized recombinant viral vectors (AAV6-MMP3 and AAV6-MMP13) per cell (TU/Cell), the resulted knockdown impacts on the expression levels of MMP3 and MMP13, and the enhancement of collagen II level, we analysed the coherence between their verified values. As shown in Figure 5, we confirmed proportional and direct relationships between the transduction units (Figure 5A), the knockdown impacts on the expressions MMP3/MMP13 (Figure 5B) and the enhancement of collagen II level (Figure 5C). To prove whether the recombinant vectors AAV6-MMP3 and AAV6-MMP13 may influence the levels of other NP relevant proteins, we analysed using ELISA the levels of additional NP critical proteins, such as IL-1β, TNF-α, ADAMTS4, ADAMTS5 and aggrecan, during the course of the study. Their levels in all treated NP cells were comparable to that in untreated NP cells throughout the test time (table 2) (*p* ≥ 0.271). Consequently, transduction of NP cells with AAV6-MMP3 or AAV6-MMP13, or a combination thereof might specifically influence the levels of MMP3, MMP13 and collagen II (*p* < 0.001) (Table 2). Age and gender showed no effect.

**TABLE 2:** Specificity of MMP3/MMP13 knockdown and collage II enhancement.

	Untreated [pg/100 µg]	AAV6-Ctrl [pg/100 µg]	AAV6-MMP3 [pg/100 µg]	AAV6-MMP13 [pg/100 µg]	AAV6-MMP3/13 [pg/100 µg]
MMP3/day 8	8,661 ± 75.5	8,685 ± 42.5	624.2 ± 13.3	8,699 ± 65.3	622.4 ± 16.4
MMP3/day 56	8,677 ± 27.5	8,710 ± 48	6,505 ± 64.9	8,671 ± 65.8	6,546 ± 87.7
MMP13/day 8	458.9 ± 3.6	459.6 ± 2.49	456.3 ± 4.74	48.62 ± 0.784	48.84 ± 0.804
MMP13/day 56	452.3 ± 2.18	454.5 ± 2.62	453.9 ± 1.57	358.8 ± 3.73	357.5 ± 3.74
Collagen II/day 8	6320 ± 54	6,312 ± 48.2	11,620 ± 112	9,552 ± 114	15,409 ± 88.8
Collagen II/day 56	6,325 ± 25.9	6,343 ± 37.9	8,255 ± 127	7,557 ± 97.5	9,376 ± 133
IL-1β/day 8	120.4 ± 1.78	120.8 ± 2.14	119.8 ± 1.71	120.6 ± 2.23	119.9 ± 1.38
IL-1β/day 56	122.3 ± 1.63	122.0 ± 2.12	121.4 ± 2	120.7 ± 1.77	121.7 ± 2.16
TNF-α/day 8	109.1 ± 2.23	108.6 ± 2.18	108.4 ± 2.61	109.4 ± 1.77	107.9 ± 1.72
TNF-α/day 56	108.8 ± 2.21	108.9 ± 1.95	107.9 ± 2.38	109 ± 2.32	109.2 ± 1.74
ADAMTS4/day 8	2,953 ± 72.6	2,979 ± 92.6	2,967 ± 96.1	2,930 ± 66.7	2,985 ± 109
ADAMTS4/day 56	2,964 ± 61.6	2,959 ± 77.4	3,001 ± 79.6	2,983 ± 49.4	3,002 ± 97.1
ADAMTS5/day 8	3,785 ± 116	3,809 ± 114	3,797 ± 137	3,794 ± 124	3,816 ± 121
ADAMTS5/day 56	3,802 ± 53.4	3,794 ± 43.1	3,795 ± 69.3	3,770 ± 57.9	3,781 ± 54.5
Aggrecan/day 8	16,321 ± 121	16,291 ± 101	16,314 ± 100	16,338 ± 120	16,304 ± 137
Aggrecan/day 56	16,315 ± 111	16,300 ± 109	16,292 ± 105	16,302 ± 104	16,336 ± 126
	Mean ± SD	Mean ± SD	Mean ± SD	Mean ± SD	Mean ± SD

To demonstrate the specificity of MMP3 and MMP13 knockdown mediated by the recombinant vectors AAV6-MMP3 and AAV6-MMP13 as well as the collage II enhancement, the levels other key NP associated proteins were analysed: such as IL-1β, TNF-α, ADAMTS4, ADAMTS5 and aggrecan. After transduction of 1 × 10^5^ NP cells with either AAV6-MMP3 or AAV6-MMP13, or a combination thereof, the cells were harvested on day 8 and day 56. Total protein was isolated and 100 μg of the protein extract from each probe was used for ELISA. Except the levels of MMP3, MMP13 and collage II, the levels of other tested NP associated proteins remained unaffected (*p* ≥ 0.271). MMP3, MMP13 and collagen II levels were affected in all transductions (*p* < 0.001), whereby the combined transduction exhibited further enhancement of collagen II. Data represent mean values of three independent experiments.

## Discussion

The causes and pathological processes of DDD are multifactorial and complex and are still not well understood. DDD is predominantly induced inside the NP. The current therapeutic measures, which are based on surgical and conservative treatments, are not able to treat the causes and the pathological processes. They might only alleviate the symptoms of the disease, without restoring the biological structure and function of the ECM. Degradation of the extracellular matrix (ECM) in NP due to the reduction of proteoglycan and collagen contents, mainly aggrecan and collagen II. This reduction exibits an imbalance in the normal physiological events for the maintenance of the ECM, namely, an imbalance in anabolic and catabolic events that can lead to reduced anabolism, enhanced catabolism or a combination thereof [31–36, 39–47]. Previously we have demonstrated in degenerative NP cells very low endogeeous expression levels of growth factors such as TGF-βs, BMPs, IGF-1, bFGF, GDF-5, PDGF and their receptors, which are important anabolic components for the stimulation of ECM synthesis. On the contrary, we recorded significantly increased endogenous expression levels of inflammatory cytokines and their receptors such as IL-1β/IL-1 R and TNF-α/TNF-α R1, where their levels increased with severity of disc degeneration. Moreover, the inflammatory cytokines activated NF-κB/MAPK signaling pathways in the analysed degenrative NP cells could lead as expected to high and increasing endogenous expression of the catabolic factors ADAMTSs and MMPs, especially ADAMTS4, ADAMT5, MMP3 and MMP13. Although these catabolic factors were counteracted by even higher and increasing expression levels of their natural endogeneous inhibitors such as TIMP-1, TIMP-2, TIMP-3 and TIMP-4, we recoded declining levels of aggrecan and collagen II with increased severity of disc degeneration [31, 32, 34–36]. This indicated that the shift of the equilibrium in the direction of ECM collapse needs in additional to the existing endogeous inhibitors also exogeous inhibitors, such as shRNAs, which can specifically target the catabolic factors like MMP3 and MMP13. Consequently, we conducted shRNA based inhibition of MMP3 and MMP13 using AAV6 as gene delivery vehicle. AAV6 mediated knockdown of MMP3 and MMP13 has not yet been attempted. The signal transductions mechanism of DDD is associated with up-regulations of the inflammatory cytokines, especiallyIL-1β and TNF-α, that exhibit vigorous proinflammatory activities. Many molecules that participate in this mechanism have been identified. The inflammasome complex containing the nod-like receptors proteins (NLRPs) is compulsively needed in this signal transduction. Activation of NLRP3 by various stimuli, such as danger-associated molecular patterns (DAMPs) of exogenous and endogenous origin or pathogen-associated molecular patterns (PAMPs) leads to the activation of IL-1β and TNF-α with their receptors IL-1R and TNFR1/TNFR2, through binding to an adapter protein known as myeloid differentiation factor 88 (MyD88). This event can recruit and phosphorylate the IL-1R associated kinase 4 (IRAK4) and TNFR associated factor 6 (TRAF6). IRAK4/TRAF6-mediated signal transduction can subsequently activate the NF-κB or MAPK pathways and deregulate the expression of various catabolic factors, like ADAMTs and MMPs, and aggravate degradation the ECM [48–53]. Hence, AAV6-RNAi-mediated inhibitions of MMP3 and MMP13 that enhanced the collagen II level could be an important treatment approach for the biological restoration of degenerative discs.

In conclusion, our findings support the biology-based regenerative treatment strategies that focus on the deceleration or reversion of DDD though inhibitions of ADAMTSs, MMPs, IL-1β and TNF-α, which are involved in the MAPK and NF-κB signaling pathways [51–53]. The overexpression of MMP3 and MMP13 worsening the decomposition of collagen II could be distinctly and stably decreased by parallel knockdown of the genes using the recombinant scAAV6-shRNA vectors. Hence, the recombinant vectors used for targeting MMP3 and MMP13 could have the potential for pharmaceutical and clinical applications as an effective and safer gene delivery system in DDD. In case of potential off-target effects regarding the clinical applications, various strategies need to be developed to overcome potential limitations that maybe associated with the recombinant AAV vectors. Admittedly, there is a reason for optimism, as the wild type AAVs are not involved in gene therapy as gene delivery vehicles. However, prospective modifications and improvements of AAV vectors can further enhance their potency, specificity and safety for gene-based therapeutic applications in DDD.

## Data Availability

The original contributions presented in the study are included in the article/supplementary material, further inquiries can be directed to the corresponding author.
